# Understanding the experiences of hikikomori through the lens of the CHIME framework: connectedness, hope and optimism, identity, meaning in life, and empowerment; systematic review

**DOI:** 10.1186/s40359-021-00605-7

**Published:** 2021-07-10

**Authors:** Jolene Y. K. Yung, Victor Wong, Grace W. K. Ho, Alex Molassiotis

**Affiliations:** 1 A130, School of Nursing, The Hong Kong Polytechnic University, Hung Hom, Kowloon, HKSAR China; 2 AAB1028, Department of Social Work, Hong Kong Baptist University, Kowloon Tong, Kowloon, HKSAR China; 3 PQ426, School of Nursing, The Hong Kong Polytechnic University, Hung Hom, Kowloon, HKSAR China; 4 GH507, School of Nursing, The Hong Kong Polytechnic University, Hung Hom, Kowloon, HKSAR China

**Keywords:** Hikikomori, Youth in social withdrawal, CHIME, Recovery, Connectedness, Hope and optimism, Identity, Meaning in life, Empowerment

## Abstract

**Background:**

Hikikomori is a phenomenon describing people who exhibit behaviors of self-secluding themselves at home for long durations of time and usually only having face-to-face social interactions with none other than family. Existing interventions for hikikomori are inconclusive and the majority are absent in using a theoretical framework to guide its components. Therefore, applicability of the psychosocial recovery framework of Connectedness, Hope and Optimism, Identity, Meaning in Life, and Empowerment (CHIME) towards hikikomori care was reviewed.

**Method:**

Five databases were searched in April 2020 with the search formula from a published systematic review on hikikomori combined with search terms specific to domains of the CHIME framework. Articles included in the review were of the English language, of all publication years, peer-reviewed, quantitative or qualitative research studies and case studies, included study designs that were observational or interventional in nature, and involved populations of socially withdrawn youth.

**Results:**

CHIME’s comprehensive structure and organized approach could guide researchers or service providers in determining areas needing assessments, measurement, and areas of focus. It is suggested that the CHIME framework is applicable after modifying a specific dimension—‘meaning of mental illness experiences’ into ‘meaning of the hikikomori experience’. Thematic overlap occurred between the domains of connectedness, identity, and meaning. Yet, additional dimensions or domains such as trust building, non-linearity, and spatiality can be included for addressing specific limitations in this application, which would help towards catering services to help hikikomori in recovery or in increasing quality-of-life of those individuals’ while entrapped in this withdrawn lifestyle.

**Conclusion:**

CHIME framework could be applicable towards hikikomori care after applying the suggested modifications. Additionally, many knowledge gaps were found in literature during this review that warrants further investigation to improve hikikomori care.

## Background

Hikikomori is a term that originated in Japan and was used as early as the 1990s [1] to describe people who socially withdraw from society or the phenomenon of their doing so [2]. Other terms, such as socially withdrawn youth [3] or hidden youth [4], have been used to describe this phenomenon in other places. Individuals with this condition seclude themselves at home for six months or longer, refrain from going to work or school, and do not maintain friendships [5]. These individuals live reclusive lifestyles and usually have face-to-face interactions only with family members [6]. Although the common definition of hikikomori refers to self-seclusion for the mentioned extended period of time, some researchers and organizations have suggested a lower threshold of three months of self-seclusion to aid in early detection and treatment [3, 6, 7]. While Hamasaki et al. [8] have suggested hikikomori as a spectrum continuum of social withdrawal (hikikomori) severity.

Five reviews of research on hikikomori were identified through a literature search. One was a systematic review and the other four were narrative reviews. The aim of the systematic review was to consolidate available research evidence on hikikomori, not to raise research questions or hypotheses [9]. Similar topics discussed in the reviews were the definition, etiology, and diagnosis of hikikomori, and interventions to treat the condition. Three definitions were mentioned in the reviews, with the major differences between them arising from the inclusion of individuals with psychiatric conditions [10, 11] or of those who might leave their home but avoid social interactions [10]. There was also further categorical differentiation into primary and secondary hikikomori, namely, those without psychiatric comorbidity and those with psychiatric comorbidities, respectively [9]. In the reviews, the following similar etiologies were described: adverse or traumatic childhood experiences, bullying, peer rejection, dysfunctional family dynamics, changes in the labor market [11, 12], and overprotective parenting styles [9, 11, 12]. The differences were: psychiatric condition [12], introverted personality, shyness, parental attachment issues, dysfunctional family dynamics, parental psychiatric conditions, poor academic performance and high expectations, technology, globalization, the Internet, the breakdown of social cohesion [13], and the overdependence of children [9]. Common issues of diagnosis mentioned in the reviews were the difficulty of differentiating hikikomori from psychiatric disorders, because those exhibiting socially isolating behaviors could potentially suffer from any one of a spectrum of psychiatric illnesses [12, 13]. There is also uncertainty over whether a psychiatric condition is the cause of hikikomori symptoms or if hikikomori leads to a psychiatric condition [10]. The following Interventions were commonly reported in the reviews: psychotherapy, pharmacological treatment, family therapy, nidotherapy, milieu therapy with the provision of a safe environment for hikikomori, support groups with the avoidance of labeling, rigid schedules, or categorization of role identity [9, 12, 13]. Less commonly reported were the following interventions: group therapy, horse-assisted therapy, communal cooking, online platforms [13], Chinese medicine, narrative therapy, naikan therapy, and engagement with social workers [9]. The following are additional interventions not mentioned in the reviews: animal-assisted therapy [14], jogging therapy [15], and the online mobile game Pokémon Go [16].

Many current interventions in hikikomori care brought up in case reports seem to lack a focal factor to target in order to achieve a recovery. The majority also fail to use a theoretical framework to guide the components of the intervention. It would be beneficial to apply a psychosocial recovery model to the task of developing methods of caring for hikikomori because such a model provides a comprehensive structure and an organized approach to guide a researcher or service provider in determining what areas need to be assessed or measured, or in identifying areas that could be focused on for care [17]. To the best of our knowledge, in only one study [18] has a psychological recovery model or framework for hikikomori care been applied. In their study, Yokoyama et al. [18] combined concepts from dialectic behavior therapy and from the mental health recovery model used by Mental Health America to design online modules for hikikomori, which uses elements of self-realization, caring for oneself, acquiring change, and future planning in the intervention. Other psychosocial recovery frameworks have not been used in hikikomori interventions. Saito [19] has proposed conceptual models on the power operates in the “hikikomori system” and the vicious circles preventing treatment for hikikomori; however, they were not focusing on psychosocial recovery. After reviewing different psychosocial recovery frameworks such as the Recovery Model [20], Psychosocial Rehabilitation Model [21], Strength Based Model [22], Coach-based Model [23], and the CHIME framework for personal recovery [24], it was concluded that all of the frameworks had something beneficial to offer for hikikomori care, such as a non-linear approach, a focus on the positive attributes of an individual or holistic care. However, it seems most fitting to apply the CHIME framework for personal recovery to hikikomori care because of the following two reasons. First, the framework was synthesized for psychosocial recovery and hikikomori are in need of psychosocial recovery from a behavioral and etiological perspective. Second, some domains and dimensions of the framework shed light on what hikikomori lack, such as connectedness, identity, or meaningful social roles, which provide accuracy in targeting specific areas requiring re-establishing for hikikomori. There is awareness that the CHIME framework has been designed for personal recovery in the area of mental health; therefore, some may hypothesize that it may be more applicable to people who have experienced mental health challenges; whereas young people with experience of primary social withdrawal may not have been diagnosed with any mental health issues or exhibited any mental health syndromes. However, the domains of the CHIME framework seem broad and encompassing which may possibly extend its application to individuals without psychiatric disorders but in need of psychosocial recovery.

### The CHIME framework

The CHIME framework for personal recovery was first synthesized by Leamy et al. [24] from a systematic review of 87 articles on frameworks used for personal recovery in mental health. It is the most comprehensive depiction of the recovery process to date [25]. This framework is versatile and has been used in studies as wide-ranging as those on cultural diversity and depression [25], and art therapy for mental health recovery [26]. The CHIME framework consists of five domains, i.e., connectedness, hope and optimism, identity, meaning in life, and empowerment. In addition, each domain contains specific dimensions.

Connectedness refers to the connection with peers, relationships, being part of the community, and receiving support from peers and others [24]. Hope and optimism refer to a belief in recovery, the motivation to change, having hope-inspiring relationships, thinking positively and valuing success, and having dreams and aspirations [24]. Identity encompasses the dimension of identity, rebuilding or redefining a positive sense of identity, and overcoming stigma [24]. Dimensions of identity have been further explained by the research team as the view that an individual can have multiple identities pertaining not only to their medical diagnosis but also including the aspects of culture, ethnicity, and sexual identity [27]. For hikikomori, much focus is placed on a status-driven or non-status driven identity. The former refers to the status of a student, worker, or trainee, while the latter may refer to, but is not limited to, the status of social activist, serious leisure devotee, volunteer, carer, and others. Hikikomori may have the identity of not being in education, training, or employment (NEET). However, it should be noted that not all NEET can be considered hikikomori, as some NEET have an active social life [6]. In the third domain of the CHIME model of meaning in life, the dimensions are: meaning of mental illness experiences, spirituality, quality of life, meaningful social roles and goals, and rebuilding life [24]. The meaning of mental illness experiences is described as finding understanding or meaning from the illness experience itself [25, 27]. If this domain is to be applied to the hikikomori population, this dimension could be replaced by the meaning of the hikikomori experience, as not all hikikomori have a comorbidity of mental illness. The last domain, empowerment, includes the dimensions of personal responsibility, control over life, and focusing on strengths [24].

As mentioned previously, the CHIME framework was described as the most comprehensive of recovery processes [25] and has domains or dimensions that match deficits in hikikomori; hence, the CHIME framework can be applied to hikikomori care. Therefore, the aim of this review was to investigate the applicability of the CHIME framework to the hikikomori population to understand their life experiences and the phenomenon. The objectives of this review were: (1) To identify studies on the hikikomori population in relation to each domain of the CHIME Framework; (2) To synthesize the identified studies and apply them to each domain of the CHIME framework; (3) To determine if the identified studies would fit into the domains of the CHIME Framework; and (4) To identify whether there are any dimensions in the CHIME Framework in which studies on the hikikomori population are lacking, which may indicate a knowledge gap. The authors hypothesize that the CHIME framework would provide an encompassing understanding of the hikikomori life experience or hikikomori phenomenon, which would be evidenced by the presence of hikikomori literature being found related to the domains of the framework and the literature would give a depiction of the life experiences of individuals with a hikikomori lifestyle or the hikikomori phenomenon. If the framework were not applicable to hikikomori, then there would been an absence of literature or a minute amount of literature found. In case this scenario happens, the domains of CHIME would be considered inapplicable to understanding the hikikomori phenomenon.

## Methodology

The reporting of this review follows the guidelines of the Preferred Reporting Items for Systematic Reviews and Meta-Analysis (PRISMA) [28]. A search for academic peer-reviewed articles was conducted and ended on April 30, 2020, using five databases: CINAHL, PubMed, ProQuest, Science Direct, and Web of Science. The search terms were “hikikomori” OR “socially withdrawn youth” OR “youth social withdrawal” OR “severe social withdrawal” OR “acute social withdrawal” OR “protracted social withdrawal” OR “prolonged social withdrawal” OR “primary social withdrawal” OR “hidden youth”, according to Li and Wong [7]. In addition, search terms specific to each domain of the CHIME framework were incorporated in the search, e.g., “spirituality” OR “quality of life” OR “relationships” OR “hope” OR “belief in recovery” OR “empowerment” OR “strength-based” OR “focusing upon strength” OR “control over life” OR “personal responsibility”, and others. Table 1 presents an example of the search strategy.

**Table 1:** Search Terms for PubMed

*Domain of connectedness*
(“hikikomori” OR “socially withdrawn youth” OR “youth social withdrawal” OR “severe social withdrawal” OR “acute social withdrawal” OR “protracted social withdrawal” OR “prolonged social withdrawal” OR “primary social withdrawal” OR “hidden youth”) AND (connectedness OR relationships OR community OR peers OR friends OR friendship)
*Domain of hope and optimism*
(“hikikomori” OR “socially withdrawn youth” OR “youth social withdrawal” OR “severe social withdrawal” OR “acute social withdrawal” OR “protracted social withdrawal” OR “prolonged social withdrawal” OR “primary social withdrawal” OR “hidden youth”) AND (hope OR optimism OR “belief in recovery” OR “motivation to change” OR “positive thinking” OR “valuing success” OR dreams OR aspirations)
*Domain of identity*
(“hikikomori” OR “socially withdrawn youth” OR “youth social withdrawal” OR “severe social withdrawal” OR “acute social withdrawal” OR “protracted social withdrawal” OR “prolonged social withdrawal” OR “primary social withdrawal” OR “hidden youth”) AND (identity OR gender OR “sexual orientation” OR culture OR “dimensions of identity” OR “rebuilding positive identity” OR “redefining positive identity” OR stigma)
*Domain of meaning in life*
(“hikikomori” OR “socially withdrawn youth” OR “youth social withdrawal” OR “severe social withdrawal” OR “acute social withdrawal” OR “protracted social withdrawal” OR “prolonged social withdrawal” OR “primary social withdrawal” OR “hidden youth”) AND (“meaning in life” OR “meaning of mental illness” OR “mental illness experience” OR “meaning of experience” OR “social roles” OR “social goals” OR “rebuilding life” OR spirituality OR “quality of life”)
*Domain of empowerment*
(“hikikomori” OR “socially withdrawn youth” OR “youth social withdrawal” OR “severe social withdrawal” OR “acute social withdrawal” OR “protracted social withdrawal” OR “prolonged social withdrawal” OR “primary social withdrawal” OR “hidden youth”) AND (empowerment OR “strength-based” OR “focusing upon strength” OR “control over life” OR “personal responsibility”)

**Fig. 1:**
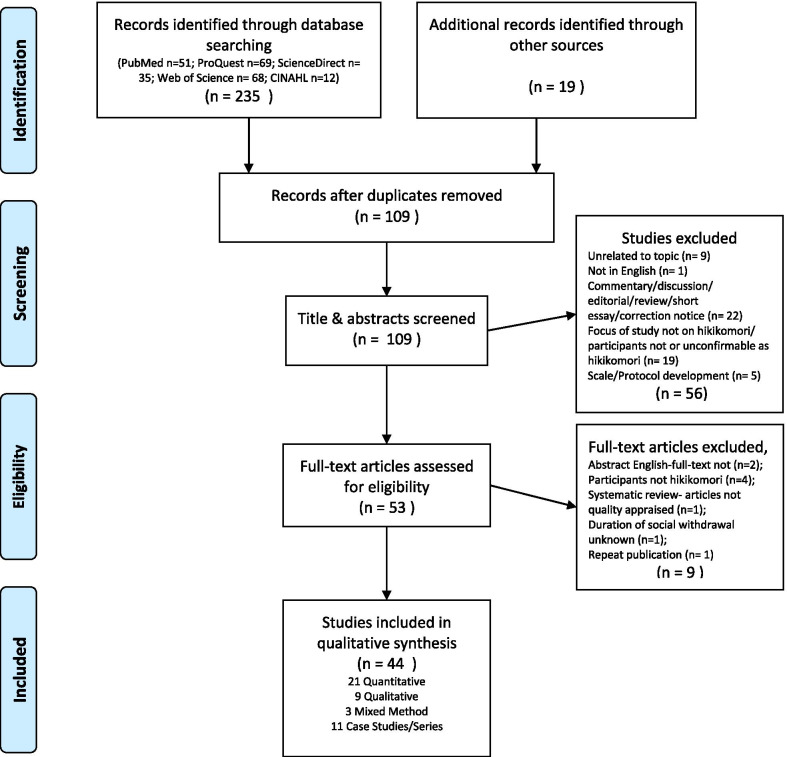
PRISMA flow diagram of search details through different phases

Articles included in this review were in the English language, of all publication years, peer-reviewed, consisted of quantitative or qualitative research studies and case studies, included study designs that were observational or interventional in nature, and involved populations of socially withdrawn youth with a minimum duration of social withdrawal of three months. Articles that did not fit this criterion or that were commentaries, discussion papers, conference abstracts, letters to the editor, or reviews, were excluded from this review. And articles that did not relate conceptually with the elements or topics within the CHIME framework were excluded from this review; while articles considered related were included. For example in the domain of connectedness, studies containing information about the social ties of hikikomori with their peers would reflect the concept of connectedness; therefore, would be included. The initial search yielded a total of 235 publications, with 51 in PubMed, 69 in ProQuest, 35 in ScienceDirect, 68 in Web of Science, and 12 from CINAHL. Titles and abstracts were screened by two reviewers and disagreements were discussed until an agreement was reached. Details of the selection and exclusion processes are displayed on the PRISMA flow chart in Fig. 1. After the primary search, reference lists of the selected articles and authors with frequent publications of hikikomori research were reviewed to identify any additional articles fitting the inclusion criteria and related to both hikikomori and elements within the CHIME framework. 19 articles were included from the hand search. The selection of full text articles were reviewed by all members of the research team and disagreements were discussed amongst all members. After quality appraisal of articles, data was extracted from the included articles relating to the life experiences of hikikomori or phenomena in accordance to the specific domains of CHIME for narrative synthesis, refer to Table 2. Extracted data was grouped in themes that were relevant to each other, data was summarized within the themes; comparisons were made either between the data or with other literature of the relevant topic depending on the availability of literature.

**Table 2:** All studies included in the review

Author(s) (year)	Participants (*N*)	Mean age, ethnicity, and gender (mean years ± SD), (*Male vs Female)*	Instruments used	Key findings
*Intervention studies*				
Lee et al. [3]	Socially Withdrawn Youth (*N* = 41), Middle/High School Students (*N* = 239)	16.4 ± 3.5, 16.3 ± 1.5 Korean 31, 10	Only data on clinical withdrawal characteristics were extracted Global Assessment Functioning Scores (GAF) (Continuous scale; scored 0–100) Higher score = Higher level of day-to-day functioning Score of 41–50 = any serious impairment in school/work/social functioning Score of 51–60 = moderate difficulty in school/work/social functioning	Minimum duration of social withdrawal identified as 3 months Internet usage (Mean hours ± SD): 5.20 ± 3.40 Overall GAF Scores for all participants before and after the home visitation psychotherapy intervention (Mean ± SD): Pre 44.6 ± 11.1 to post 53.4 ± 13.2, p < 0.001 48.8% of socially withdrawn youth showed no change in GAF scores post intervention
Malagón-Amor et al. [39]	Socially Withdrawn Adults (*N* = 190)	39.1 ± 18.1 Spanish 136, 54	Self-developed questionnaire assessing social network of relationships Sociodemographic data and clinical history were extracted from the patients’ files	No statistically significant difference in social networks at the following time points: 4 months: p = 0.200 8 months: p = 0.947 12 months: p = 0.991
Chan [75]	Hidden Youth (*N* = 502)	12–21 years Chinese 384, 118	Self-developed questionnaire assessing participation in therapeutic activities Empowerment Scale per Rogers et al. (28-Likert items; scored 20–80) Higher score = levels of empowerment Psychological Capital Questionnaire (24-Likert items; scored 21–126) Higher score = higher psychological well-being	Relationships by Correlations and P-values of Hikikomori Play Therapy to: Empowerment = 0.59, p = 0.0000 Psychosocial well-being = 0.60, p = 0.0000 Level 3 Hierarchical Regression: Empowerment = 9.56, p = 0.0000 Play Therapy = 0.64, p = no statistical significance No scale scores were reported
Law et al. [74]	Youth (*N* = 373)	12–29 years Chinese 171, 202	Used a proxy checklist as per Uchida and Norasakkunit to measure social withdrawal (4-item yes/no answers)	Decrease in social withdrawal by 4.61% No scale scores reported for socially withdrawn youth
Yokoyama et al. [18]	Hikikomori (*N* = 5)	16–35 years Japanese 1, 4	Self-developed survey administered post intervention	C-BED intervention: Decrease in anxiety, increase in willingness to participate post intervention No measurement scales used in the study
*Cross-sectional studies*				
Koyama et al. [62]	Hikikomori (*N* = 19), Community Residence (*N* = 1641)	20–49 years Japanese 14, 5	Face-to-face household survey assessing sociodemographic data, hikikomori symptoms, and psychiatric history	Demographic data of males and females from Japan, age 20–49
Kondo et al. [40]	Hikikomori (N = 337)	24.2 ± 5.4 Japanese 252, 85	Psychiatrists elicited information on sociodemographic data and psychiatric history during consultations	Education level (%): Junior High: 34.4% High School: 39.5% University/College: 25.5% Demographic data of males and females from Japan, with a mean age of 24
Nagata et al. [64]	Hikikomori (*N* = 27), Patients with Social Anxiety Disorder (*N* = 114)	27.4 ± 7.5 Japanese 12, 15	Information on sociodemographic data and psychiatric history were elicited from retrospective clinical data	Sociodemographic data on the distribution of males and females from Japan, with a mean age of 27, and with 11.7 ± 1.7 years of education; however, it was unclear whether the data on education included the preschool years
Umeda and Kawakami [63]	Hikikomori (*N* = 15), Community Residence (*N* = 693)	36.3 ± 11.1, 30.1 ± 8.6 Japanese 10, 5	Face-to-face population-based survey conducted in metropolitan, urban, and rural areas of Japan assessing sociodemographic data, hikikomori symptoms, psychological history, childhood social class, parental psychological history, and childrearing practices	Education level (%): Junior High: 3.8% High School: 71.4% University/College: 24.8% Demographic data on males and females from Japan, with a mean age 36
Krieg and Dickie [37]	Hikikomori (*N* = 24), University Students (*N* = 59)	22.84 years, 20.59 years Japanese 14, 10	Trait Shyness Scale (16-Likert items; scored 16–80) Maternal Attachment Scale Insecure/avoidant attachment subscale (8-Likert items; scored 1–4) Insecure/ambivalent attachment subscale (7-Likert items; scored 1–4) Parental Rejecting Behavior Scale Ignoring subscale (1-Likert item; scored 1–7) Threaten subscale (1-Likert item; scored 1–7) Lock-out subscale (1-Likert; scored 1–7) Composite score (Sum of subscale scores) Peer Rejection Scale (1-Likert item; scored 1–8) Maladjustment to School Scale Difficulty with adjusting to peer group work (1-Likert item; scored 1–7) All Japanese versions. Higher score = higher disturbance/intensity	Hikikomori vs University students for various scales (Mean ± SD): Shyness score: 52.83 ± 12.27 vs 46.89 ± 9.76 Maternal attachment Avoidance score: 2.21 ± 0.70 vs 2.09 ± 0.71 Ambivalence score: 2.08 ± 0.75 vs 1.51 ± 0.51 Parental rejection score Locking out score: 3.58 ± 2.21 vs 3.21 ± 2.37 Threatening loss of relationship score: 3.71 ± 1.94 vs 2.05 ± 1.61 Ignoring score: 3.00 ± 2.09 vs 2.15 ± 1.57 Composite score: 10.29 ± 4.44 vs 7.41 ± 4.02 Peer rejection: 3.85 ± 2.31 vs 2.41 ± 2.15 Maladjustment to school: 4.50 ± 1.62 vs 3.20 ± 1.85 Hikikomori Correlation and P-values: Shyness: 0.285, p < 0.05 Ambivalent maternal attachment: 0.400, p < 0.01 Adjustment to middle school: 0.391, p < 0.01 Parental rejection: 0.302, p < 0.01 Threatened loss of relationship: 0.405, p < 0.01 Ignoring: 0.219, p < 0.05 Peer rejection: 0.287, p < 0.01
Chan and Lo [60]	Hidden Youth (*N* = 588)	12–30 years Chinese 373, 215	World Health Organization Quality of Life scale-brief (WHOQOL-BREF) (28-Likert item; scoring 0–112) Higher score = Higher Quality of Life	Hidden Youth QOL Correlations and P-values Low level of social withdrawal = 0.550, p = 0.0000 High level of social withdrawal = -0.850, p = 0.0000 WHOQOL-BREF scores not reported
Malagón-Amor et al. [59]	Hikikomori (*N* = 164)	40.0 ± 18.3 Spanish 121, 43	Information on sociodemographic data and psychiatric history were elicited from clinical data on consultations	Sociodemographic data on the distribution of males and females from Spain
Uchida and Norasakkunkit [58]	Hikikomori (*N* = 114), NEET (*N* = 86), Employed Adults (*N* = 7525)	20–39 years Japanese –	NEET Hikikomori Risk Scale Unclear ambitions (2-items; scoring 0–12) Higher score = Higher level of unclear ambitions	Hikikomori vs NEET vs Employed Adults (Mean ± SD) Unclear ambitions: 5.48 ± 1.60 vs 5.05 ± 1.41 vs 4.26 ± 1.37
Frankova [61]	Hikikomori (*N* = 35), Control group (*N* = 28)	18–40 years Ukrainian 14, 21	Chaban Quality of Life Scale (10- item scale, scoring not reported, newly developed scale from Ukraine) Higher score = Higher Quality of Life	Hikikomori (with and without psychiatric comorbidities) vs Control group 11.7 ± 2.70; p = 0.001 and 13.7 ± 3.3; p = 0.001, vs 19.3 ± 3.50
Chauliac et al. [38]	Socially Withdrawn Youth (*N* = 66)	23.2 ± 4.75 French 53, 13	Data on clinical withdrawal characteristics extracted from patient records (Frequency only)	Relationships maintained (%) with: Family: 63% Friends: 8% Family and friends: 8% Family and sentimental/school: 3% None: 19% Going on outings (%): Alone: 38% Accompanied: 35% None: 27% Poor hygiene (%): Yes: 33% No: 67%
Yuen et al. [41]	Hikikomori (*N* = 104)	19.02 ± 3.62 Chinese 62, 42	Self-developed questionnaire to assess sociodemographic data, daily activities, and health history Modified Berkman–Syme Social Network Index (7 yes/no items; scored 0–7) Higher score = higher connectedness	Types of Daily activities (Mean hours ± SD): Sleeping: 7.83 ± 1.99 Computer use: 5.09 ± 4.97 Tablet/mobile use: 3.11 ± 5.03 Eating: 1.90 ± 1.03 Reading comics/animations: 0.95 ± 2.38 Watching television: 0.90 ± 1.13 Other reading: 0.65 ± 2.09 Idling/Facing the wall: 0.40 ± 0.99 Social Network Index (Mean ± SD): 2.79 ± 1.80
Yong and Nomura [43]	Hikikomori (*N* = 58), Non-Hikikomori (*N* = 3024)	15–39 years Japanese 38, 20	Interpersonal difficulties assessed by 4 yes/no questions: Fear of meeting others (Q1) Anxiety about meeting familiar people (Q2) Anxiety about people’s impression of oneself (Q3) Cannot blend into groups (Q4)	Hikikomori vs non-hikikomori (% Interpersonal difficulties; p-value): Overall = 74.1 vs 36.0; < 0.001 Q1 = 36.2 vs 8.1; < 0.001 Q2 = 48.3 vs 7.1; < 0.001 Q3 = 51.7 vs 28.3; < 0.001 Q4 = 53.4 vs 14.6; < 0.001 Social class of hikikomori (%) Upper: 3.4% Middle: 77.6% Lower: 19.0%
Wu et al. [42]	Socially Withdrawn Adults (*N* = 168), Non-Socially Withdrawn Adults (*N* = 258)	28.82 ± 0.60 Taiwanese 78, 90	Self-developed online survey assessing sociodemographic data, social withdrawal behavior, and psychiatric history	Education: Bachelor Level: 90.0% Lived in areas of (%): Low income: 72.6% Middle income: 18.5% High income: 3.6%
*Longitudinal studies*				
Yuen et al. [35]	Hikikomori (*N* = 104)	19.02 ± 3.62 Chinese 62, 42	Chinese Interpersonal Support Evaluation List (ISEL)- Short version (12-Likert items; scored 0–48) Appraisal Support subscale (4-Likert items; scored 0–12) Belonging Support subscale (4-Likert items; scored 0–12) Tangible Support subscale (4-Likert items; scored 0–12) Self-Esteem Support subscale (4-Likert items; scored 0–12) Higher score = higher social support Modified Berkman–Syme Social Network Index (7 yes/no items; scored 0–7) Higher score = higher connectedness	ISEL (Mean ± SD): Time points 1–3: 24.60 ± 6.30, 24.63 ± 5.99, 24.75 ± 6.89 Appraisal Support: 6.81 ± 2.16, 6.90 ± 1.87, 7.11 ± 2.18 Tangible Support: 6.20 ± 1.71, 6.29 ± 1.53, 6.20 ± 1.73 Belonging Support: 6.00 ± 2.45, 5.89 ± 2.53, 6.05 ± 2.44 Self-Esteem Support: 5.59 ± 2.08, 5.55 ± 2.14, 5.38 ± 2.39 SNI (Mean ± SD): Time points 1–3: 2.79 ± 1.80, 2.93 ± 2.06, to 3.09 ± 1.87
*Pilot case–control studies*				
Katsuki et al. [65]	Hikikomori (n = 22), Non-Hikikomori (*N* = 18)	33.14 ± 9.33, 37.94 ± 8.93 Japanese 12, 10	Rorschach Comprehensive System; 10-inkblot items, scored as per Takahashi et al. for the Japanese population and scored by a clinical psychologist Form Color; higher scores indicate a higher level of passive aggressiveness, a tendency to adjust one’s emotions to the environment and people, an inclination to suppress the expression of emotions when feeling shaken in social situations SumT; total number of texture-related responses, higher scores indicate a need for and an openness to forming close emotional relationships	Hikikomori vs Non-hikikomori (Mean ± SD): Form Color: 2.50 ± 1.68 vs 1.39 ± 1.14, p = 0.037 SumT: 0.50 ± 0.67 vs 0.11 ± 0.32, p = 0.033
*Mixed-method studies*				
Chan and Lo [60]	Hidden Youth (*N* = 363)	21.11 ± 2.93 Chinese 244, 119	Self-developed survey assessing sociodemographic data Relationships with Parents Scale as per Zeng and Zeng’s (8- items; scored:?) Relationships with Siblings Scale as per Zeng and Zeng’s (7-items; scored:?) Relationships with Teachers as per Deng; Xu and Ma’s (7- items; scored:?) Relationships with Peers as per Deng; Xu and Ma’s (9- items; scored:?) Rosenberg Self-Esteem Scale (10-Likert items; scored 0–30) Higher score = higher self-esteem; < 15 = low self-esteem, ≥ 15 = high self- esteem	Self-esteem of Hidden Youth in Correlations and P-values with a good relationship with: Parents = 0.73, p = 0.0000 Siblings = 0.66, p = 0.0000 Teachers = 0.13, p < 0.05 Peers = 0.16, p < 0.05 No scale scores were reported
Chan [76]	Hidden Youth (*N* = 502)	12–24 years Chinese 384, 118	Self-developed questionnaire assessing sociodemographic data and uses of counselling services World Health Organization Quality of Life scale-brief (WHOQOL-BREF) Taiwan Version (measured well-being after receiving counselling services) Young Person’s CORE (10- Likert items; scored 0–40) Lower score = positive results Qualitative Semi-structured interviews assessing perceived advantages & usefulness of three forms of counseling	Total Quality of Life scores (Mean ± SD): Online counselling: 3.02 ± 0.43 Offline counselling: 2.52 ± 0.27 Integrated counselling: 3.74 ± 0.60 Young Person’s Core (Mean ± SD): Online counselling: 1.56 ± 1.29 Offline counselling: 0.62 ± 0.42 Integrated counselling: 2.59 ± 1.18 Interview results: online counselling offered platform for communication while offline counselling offered opportunity for mediation during conflicts between youth and their family
Chan [55]	Hidden Youth (*N* = 357)	12–30 years Chinese –	Self-constructed questionnaires assessing forms of communication and friendship levels Miller Social Intimacy Scale as per Miller & Lefcourt (17 items; scored: 17–170) Higher scores = higher levels of social intimacy Qualitative semi-structured interviews assessing how youth choose usage of forms for communication	Forms of communication used: Public text: People you only know (100%); Friends (100%); Good friends (100%); Best friends (100%) Public voice: People you only know (3.2%); Friends (49.0%); Good friends (100%); Best friends (100%) Public camera meeting: People you only know (0%); Friends (6.0%); Good friends (91.6%); Best friends (100%) Private text: People you only know (3.2%); Friends (43.0%); Good friends (100%); Best friends (100%) Private voice: People you only know (3.2%); Friends (30.0%); Good friends (100%); Best friends (100%) Private camera meeting: People you only know (0%); Friends (17.0%); Good friends (92.6%); Best friends (100%) Scores for Miller Social Intimacy Scale not reported
*Case studies/series*				
Hattori [73]	Hikikomori (*N* = 35)	21.5 years Japanese 25, 10	Clinical data extracted from clinical records and consultations	Recovery time: minimum 2 years Patient mistrust of therapist Spent 6–12 months testing the reliability of the therapist. Psychotherapy: 50% attrition rate
Sakamoto et al. [47]	Hikikomori (*N* = 1)	24 years Omani 1 Male	Clinical data extracted from a patient case	Exhibited the following behaviors: confined self at home, spent the majority of time in own room, did not engage in social relationships, reversed sleep/wake cycle, and refused contact with family Demographic data of a male from Oman, age 24
Teo [49]	Hikikomori (*N* = 1)	30 years American 1 Male	Clinical data extracted from a patient case	Exhibited the following behaviors: confined self at home, spent the majority of time in own room, and did not engage in social relationships. Poor hygiene practices & urinated/defecated in jars/bottles Demographic data of a male from the United States, age 30
Suwa et al. [51]	Hikikomori (*N* = 1)	25 years Japanese 1 Male	Clinical data extracted from a patient case	Exhibited the following behaviors: exhausted from effort to maintain relationships, inability to relate well with others, fear in entering adult society, no confidence to cope with society, felt ashamed of himself, and feared others opinions of him being unemployed
Ovejero et al. [45]	Hikikomori (*N* = 1)	25 years Spanish 1 Male	Clinical data extracted from a patient case	Exhibited the following behaviors: confined self at home, spent the majority of time in own room, and did not engage in social relationships Demographic data of a male from Spain, age 25
Teo et al. [36]	Hikikomori (*N* = 36)	18–49 years American, Japanese, Korean, & Indian 29, 7	LSNS-6 (6-Likert item, scoring 0–30) Measuring social connectedness Family subscale (3-Likert items; scored 0–15) Friends subscale (3-Likert items; scored 0–15) Total overall score > 12 = social isolation UCLA Loneliness Scale (20-Likert items, scoring 20–80) Higher score = Higher level of loneliness	LSNS-6 Scale score (Mean ± SD): Overall score: 9.7 ± 5.7 Family score: 5.4 ± 3.0 Friends score: 4.3 ± 3.5 UCLA Loneliness Scale (Mean ± SD): 55.4 ± 10.5 Demographic data on the distribution of males and females, from across four countries; education level: 56.0% hold a bachelor’s degree or above
Ranieri [46]	Hikikomori (*N* = 2)	13 years Italian 2 Females	Clinical data extracted from patient cases	Exhibited the following behaviors: confined self at home, spent the majority of time in own room, did not engage in social relationships, and refused contact with family Demographic data of two females from Italy, age 13 Psychotherapy recovery time 4 years
Kato et al. [44]	Hikikomori (*N* = 1)	39 years Japanese 1 Male	Clinical data extracted from a patient case	Exhibited the following behaviors: confined self at home, spent the majority of time in own room, did not engage in social relationships, avoided face-to-face contact with others, reversed sleep/wake cycle, and left home once a month to visit an outpatient clinic Demographic data of a male from Japan, age 39
Matsuguma et al. [77]	Hikikomori (*N* = 1)	17 years Japanese 1 Male	Rosenberg Self-Esteem Scale (10-Likert items; scored 0–30) Higher score = higher self-esteem; < 15 = low self-esteem, ≥ 15 = high self-esteem Subjective Vitality Scale (measures feelings of alertness or being energized, scoring 0–4) Higher score = higher vitality Higher score = higher vitality Kessler Psychological Distress Scale (measures anxiety and depression, scoring 10–50) Lower scores = lower distress levels	Strength-based coaching intervention pre-post scores: Rosenberg Self-Esteem Scale: 16 to 25 Subjective Vitality Scale: 1.8 to 3.4 Kessler Psychological Distress Scale: 17 to 6
Ranieri [46]	Hikikomori (*N* = 1) Mixed group	19 years – 1 Male	Clinical data extracted from a patient case	Exhibited the following behaviors: social anxiety, mistrust in parents, inability to approach the opposite gender when interested. Demographic data of a male, age 19
Silic et al. [48]	Hikikomori (*N* = 1)	24 years Croatian 1 Male	Descriptive data extracted only	Exhibited the following behaviors: confined self at home, spent the majority of time in own room, did not engage in social relationships, refused contact with family, and used furniture to block entry to room to avoid contact Demographic data of a male from Croatia, age 24
Roza et al. [72]	Hikikomori (*N* = 1)	35 years Brazilian 1 Male	Clinical data extracted from a patient case	Exhibited the following behaviors: confined self at home, spent the majority of time playing computer games, and did not engage in social relationships. Neglected self-care and hygiene. Demographic data of a male from Brazil, age 35
*Qualitative studies*				
Ogino [56]	Hikikomori (*N* = ?)	26.7 years Japanese –	Empirical data from 20 h site contact and qualitative interviewing with hikikomori, and staff and group leaders in support group	Life experiences: motivation to return to society but obstacles they could not overcome, lack a positive sense of identity; feelings of anxiety, unable to do anything, fear of failure, lacking in self-esteem or self-confidence; having lack of qualifications on resumé; difficulties to explaining themselves to people because lack of social identity; use of intervention called Free Space Wood
Kaneko [52]	Hikikomori (*N* = 1)	Mid-30’s Japanese 1 Male	Empirical data of field research and qualitative interviewing	Life experiences of: lack of trust in people
Wong and Ying [54]	Socially Withdrawn Youth (*N* = 88)	13–24 years Chinese 67, 21	Individual and focus group qualitative interviews	Life experiences: intimate relationships online, but no intention of meeting the other person; use of intervention involving social worker engagement, social workers have accompanied socially withdrawn youth to outings to providing sense of security Recommendations: process of recovery a “yo-yo process” with setbacks of reversal in progress noted; recovery needs to be at pace of youth; a non-linear process; building trust is an important stage in their recovery process; and starting where client is at
Wong [6]	Socially Withdrawn Youth (*N* = 252)	13–24 years Chinese 202, 50	Empirical data from clinical data and literature	Life experiences: face-to-face interactions only with family members, mother had no face-to-face contact with child for months; use intervention of home visiting withdrawn youth, can have sudden refusal from youth to take part in social activities/face-to-face contact during social worker reengagement Supplementary data: NEET can be considered hikikomori, but some NEET have an active social life Recommendations: home visiting requires sensitivity and awareness, recognition when client needs privacy, sensitivity to client’s surroundings provides clues in relation to hobbies/interests which can be topics for discussion during reengage of client
Wong [70]	Socially Withdrawn Youth (*N* = 30)	15–24 years Chinese –	Individual and focus group qualitative interviews	Life experience: sensitive name calling of “withdrawal guys or hidden youth”; self-secluding nature of hikikomori, the majority of care provided in the home; recommendations of “starts where the client is”
Tajan [11]	Recovered Hikikomori (*N* = 4)	29–50 years Japanese 3, 1	One-to-one qualitative interviews	Life experience: positive and inspiring relationships from meeting someone they trusted, receiving encouragement from someone they knew and liked, feeling that no one would care if they died and living like an animal
Rubinstein [71]	Hikikomori (*N* = ?)	– Japanese –	Empirical data from 50 interviews on site of support groups, hikikomori and mental illness communities with parents, children, hikikomori-related program staff and field observation	Life experience: parents desired to avoid stigma and labeling of mental illness therefore chose to call their child a hikikomori, even after receiving a psychiatric diagnosis; did not claim that the hikikomori experience was pleasant
Yong and Kaneko [57]	Hikikomori (*N* = 5) Proxy respondents *(N* = 3) Online forum participants (*N* = 160)	–	One-to-one qualitative interviews and empirical data extraction from internet forum from first person and second person experiences	Life experiences: cautious about establishing relationships over the Internet due to fear and inability to trust people, loss of trust in people, low self-esteem, feelings of hopelessness for future, having non-competitiveness, ineffective communication, lack a positive sense of identity and confidence, negative appraisals from others and self, negative thinking or thoughts of self, fear of social interactions, unable to secure a job, feelings of inadequacy or incompetence, complains society too demanding and unfair, felt unable to do anything, exhibits confinement at home, little social contact
Li and Wong [53]	Socially Withdrawn Youth (*N* = 30)	14–29 years Chinese 22, 8	One-to-one semi-structured qualitative interviews	Life experiences: lifestyle centered on confinement at home, with little social contact, losing touch with the outside world, lacking face-to-face contact with others, having no peer relationships, unable to get along with others, lack of trust in people, experienced a traumatic life event, use the Internet to find positive self-identity and positive and inspiring, having negative feelings or thoughts, boredom, sense of no longer stand staying at home, a loss of interest in computer games, some enjoying their seclusion and feeling freed from restraints and timelines

*vs* versus, *N* Total number of participants, *h* hours, *SD* Standard deviation, *NEET* not in employment, education, or training, *QOL* quality of life, *C-BED* community based enterprise component

### Quality appraisal

All included articles were assessed for biases and rigor in methodology using the Joanna Briggs Institute (JBI) Critical Appraisal Checklist tool [29–33] and Mixed Method Appraisal Tool (MMAT) by the Department of Medicine of the McGill University [34]; and were of high quality, refer to Table 3. The JBI contains separate checklists for quasi-experimental (9 criteria), case–control (10 criteria), cohort (11 criteria), analytical and prevalence cross-sectional studies (8 and 9 criteria), case reports or series (8 and 10 criteria), and qualitative (10 criteria). Each component of the checklist can be rated as yes, no, unclear, or not applicable. The MMAT contains six sections in the checklist; for mixed method studies only four sections are used (initial screening section, followed by sections 1; 2, 3, or 4; and 5) with ratings of yes, no, or can’t tell.

**Table 3:** Quality appraisal of articles included in review with JBI and MMAT tools

Criteria for assessment in JBI									
JBI for quasi-experimental									
Study	Is it clear in the study what is the ‘cause’ and what is the ‘effect’?	Were the participants included in any comparisons similar?	Were the participants included in any comparisons receiving similar treatment/care, other than the exposure or intervention of interest?	Was there a control group?	Were there multiple measurements of the outcome both pre and post the intervention/exposure?	Was follow up complete and if not, were differences between groups in terms of their follow up adequately described and analyzed?	Were the outcomes of participants included in any comparisons measured in the same way?	Were outcomes measured in a reliable way?	Was appropriate statistical analysis used?
Lee et al. [13]	✓	✓	✓	✓	✓	✓	✓	?	✓
Malagón-Amor et al. [39]	✓	✓	✓	X	✓	✓	✓	?	✓
Law et al. [74]	✓	✓	✓	✓	✓	✓	X	?	✓
Yokoyama et al. [18]	✓	✓	✓	X	✓	?	✓	?	N/A
Chan [75]	✓	✓	✓	X	✓	?	✓	?	✓

JBI for case control										
Study	Were the groups comparable other than the presence of disease in cases or the absence of disease in controls?	Were the groups comparable other than the presence of disease in cases or the absence of disease in controls?	Were the same criteria used for identification of cases and controls?	Was exposure measured in a standard, valid and reliable way?	Was exposure measured in the same way for cases and controls?	Were confounding factors identified?	Were strategies to deal with confounding factors stated?	Were outcomes assessed in a standard, valid and reliable way for cases and controls?	Was the exposure period of interest long enough to be meaningful?	Was appropriate statistical analysis used?
Katsuki et al. [65]	✓	?	✓	✓	✓	X	X	✓	N/A	✓

JBI for analytical cohort											
Study	Were the two groups similar and recruited from the same population?	Were the exposures measured similarly to assign people to both exposed and unexposed groups?	Was the exposure measured in a valid and reliable way?	Were confounding factors identified?	Were strategies to deal with confounding factors stated?	Were the groups/participants free of the outcome at the start of the study?	Were the outcomes measured in a valid and reliable way?	Was the follow up time reported and sufficient to be long enough for outcomes to occur?	Was follow up complete, and if not, were the reasons to loss to follow up described and explored?	Were strategies to address incomplete follow up utilized?	Was appropriate statistical analysis used?
Yuen et al. [35]	✓	✓	✓	X	X	✓	✓	✓	✓	✓	✓

JBI for analytical cross-sectional								
Study	Were the criteria for inclusion in the sample clearly defined?	Were the study subjects and the setting described in detail?	Was the exposure measured in a valid and reliable way?	Were objective, standard criteria used for measurement of the condition?	Were confounding factors identified?	Were strategies to deal with confounding factors stated?	Were the outcomes measured in a valid and reliable way?	Was appropriate statistical analysis used?
Kondo et al. [40]	✓	✓	✓	✓	X	X	✓	✓
Krieg and Dickie [37]	✓	✓	✓	✓	X	X	✓	✓
Nagata et al. [64]	✓	✓	✓	✓	X	X	✓	✓
Chan and Lo [4]	✓	✓	✓	✓	X	X	✓	✓
Uchida and Norasakkunkit [58]	✓	✓	✓	✓	X	X	✓	✓
Umeda et al. [63]	✓	✓	✓	✓	✓	✓	✓	✓
Yuen et al. [41]	✓	✓	✓	✓	X	X	✓	✓
Yong and Nomura [43]	✓	✓	✓	✓	X	X	✓	✓
Wu et al. [42]	✓	✓	✓	✓	?	?	✓	✓

JBI for prevalence cross-sectional									
Study	Was the sample frame appropriate to address the target population?	Were study participants sampled in an appropriate way?	Was the sample size adequate?	Were the study subjects and the setting described in detail?	Was the data analysis conducted with sufficient coverage of the identified sample?	Were valid methods used for the identification of the condition?	Was the condition measured in a standard, reliable way for all participants?	Was there appropriate statistical analysis?	Was the response rate adequate, and if not, was the low response rate managed appropriately?
Koyama et al. [62]	✓	✓	✓	✓	✓	✓	✓	✓	✓
Malagón-Amor et al. [59]	✓	✓	✓	✓	✓	✓	✓	✓	✓
Chauliac et al. [38]	✓	✓	N/A	✓	✓	✓	✓	✓	N/A
Frankova [61]	✓	✓	✓	✓	✓	✓	✓	✓	✓

JBI for case series										
Study	Were there clear criteria for inclusion in the case series?	Was the condition measured in a standard, reliable way for all participants included in the case series?	Were valid methods used for identification of the condition for all participants included in the case series?	Did the case series have consecutive inclusion of participants?	Did the case series have complete inclusion of participants?	Was there clear reporting of the demographics of the participants in the study?	Was there clear reporting of clinical information of the participants?	Were the outcomes or follow up results of cases clearly reported?	Was there clear reporting of the presenting site(s)/clinic(s) demographic information?	Was statistical analysis appropriate?
Teo et al. [36]	✓	✓	✓	✓	✓	✓	?	✓	✓	✓

JBI for Case Reports/Studies								
Study	Were patient’s demographic characteristics clearly described?	Was the patient’s history clearly described and presented as a timeline?	Was the current clinical condition of the patient on presentation clearly described?	Were diagnostic tests or assessment methods and the results clearly described?	Was the intervention(s) or treatment procedure(s) clearly described?	Was the post-intervention clinical condition clearly described?	Were adverse events (harms) or unanticipated events identified and described?	Does the case report provide takeaway lessons?
Sakamoto et al. [47]	✓	✓	✓	✓	?	✓	✓	✓
Hattori [73]	✓	✓	✓	✓	✓	✓	X	✓
Suwa and Suzuki [51]	✓	✓	✓	X	X	✓	✓	X
Teo [49]	?	✓	✓	✓	✓	✓	X	✓
Overjero et al. (2014)	X	✓	✓	✓	✓	✓	X	✓
Ranieri [46]	✓	✓	✓	X	?	✓	X	✓
Kato et al. [44]	X	✓	✓	✓	?	✓	✓	X
Ranieri [46]	?	✓	✓	?	?	✓	✓	✓
Matsuguma et al. [77]	✓	✓	✓	✓	?	✓	X	✓
Silić et al. (2019)	✓	✓	✓	✓	✓	✓	X	✓
Roza et al. [72]	X	✓	✓	✓	✓	✓	X	✓

JBI for Qualitative										
Study	Is there congruity between the stated philosophical perspective and the research methodology?	Is there congruity between the research methodology and the research question or objectives?	Is there congruity between the research methodology and the methods used to collect data?	Is there congruity between the research methodology and the representation and analysis of data?	Is there congruity between the research methodology and the interpretation of results?	Is there a statement locating the researcher culturally or theoretically?	Is the influence of the researcher on the research, and vice- versa, addressed?	Are participants, and their voices, adequately represented?	Is the research ethical according to current criteria or, for recent studies, and is there evidence of ethical approval by an appropriate body?	Do the conclusions drawn in the research report flow from the analysis, or interpretation, of the data?
Ogino [56]	✓	✓	✓	✓	✓	✓	?	✓	X	✓
Kaneko [52]	✓	✓	✓	✓	✓	✓	?	✓	?	✓
Wong and Ying [54]	✓	✓	✓	✓	✓	✓	?	✓	?	✓
Wong [6]	✓	✓	✓	✓	✓	✓	?	?	?	✓
Wong [70]	✓	✓	✓	✓	✓	✓	?	✓	✓	✓
Tajan [11]	✓	✓	✓	✓	✓	✓	?	✓	?	✓
Rubinstein [71]	✓	✓	✓	✓	✓	✓	✓	?	✓	✓
Yong and Kaneko [57]	✓	✓	✓	✓	✓	✓	✓	✓	✓	✓
Li and Wong [53]	✓	✓	✓	✓	✓	✓	✓	✓	?	✓

Criteria for assessment for MMAT																	
MMAT for mixed method																	
Study	Are there clear research questions?	Do the collected data allow to address the research questions?	Is the qualitative approach appropriate to answer the research question?	Are the qualitative data collection methods adequate to address the research question?	Are the findings adequately derived from the data?	Is the interpretation of results sufficiently substantiated by data?	Is there coherence between qualitative data sources, collection, analysis and interpretation?	Are the participants representative of the target population?	Are measurements appropriate regarding both the outcome and exposure/intervention?	Are there complete outcome data?	Are the confounders accounted for in the design and analysis?	During the study period, is the intervention/exposure administered as intended?	Is there an adequate rationale for using a mixed methods design to address the research question?	Are the different components of the study effectively integrated to answer the research question?	Are the results adequately brought together into overall interpretations?	Are divergences and inconsistencies between quantitative and qualitative results adequately addressed?	Do the different components of the study adhere to the quality criteria of each tradition of the methods involved?
Chan and Lo [60]	✓	✓	✓	✓	✓	✓	✓	✓	✓	✓	X	✓	✓	✓	✓	✓	✓
Chan [76]	✓	✓	✓	✓	✓	✓	✓	✓	✓	✓	X	✓	✓	✓	✓	✓	✓
Chan [55]	✓	✓	✓	✓	✓	✓	✓	✓	✓	✓	X	✓	✓	✓	✓	✓	✓

✓, Yes; X, No; Question Mark (?) , Unclear or Can’t tell; N/A, Not applicable; JBI, Joanne Brigg’s Institute Appraisal Tool; MMAT, Mixed Method Appraisal Tool.

**Table 4:** Applicability of the CHIME framework to assessing hikikomori care

Domain	Applicable	Not studied
Connectedness		
Support by peers or others	✓	
Relationships	✓	
Part of community	✓	
Hope and optimism		
Belief in recovery		✓
Motivation to change	✓	
Hope-inspiring relationships	✓	
Positive thinking and valuing success	✓	
Having dreams and aspirations		✓
Identity		
Dimensions of identity:		
Race	✓	
Gender	✓	
Social class	✓	
Culture	✓	
Religion		✓
Sexual orientation	✓	
Personal attributes / characteristics / identity	✓	
Rebuilding/redefining a positive sense of identity	✓	
Overcoming stigma		✓
Meaning in life		
Meaning of hikikomori experiences:		
Duration of social withdrawal	✓	
Activities of daily life	✓	
Feelings & Thoughts	✓	
Spirituality		✓
Quality of life	✓	
Meaningful life and social roles/goals	✓	
Rebuilding life	✓	
Empowerment		
Personal responsibility		✓
Control in life		✓
Focusing upon strengths	✓	

## Results

A total of 44 studies were identified, 21 of which were quantitative, 9 qualitative, 3 mixed methods, and 11 case studies or series. They were published between the years 2004 and 2020. The studies came from various countries, although the majority were conducted in Japan. All of studies are listed and described in Table 2. Below, the studies are presented in accordance with each domain of the CHIME framework.

### Domain of connectedness

Connectedness refers to the link with peers, relationships, being part of the community, and receiving support from peers and others in the CHIME framework [24]. In this domain 23 articles were found related to connectedness in hikikomori. These articles included two interventional studies; six cross-sectional studies; one mixed-method studies; one longitudinal study; nine case studies, reports or case series; and four qualitative studies.

Two studies that measured connectedness in hikikomori reported low levels of connectedness. One study using the Modified Berkman–Syme Social Network Index (SNI), which measures social ties and involvement in relationships, reported the low SNI score of 2.79 ± 1.80 out of 7 (Table 2) in hikikomori [35]. This was also reflected in another study using a different measurement tool, in which low scores for social connectedness (9.7 ± 5.7 out of 30.0 in the Lubben Social Network Scale (LSNS)-6 questionnaire) were also found (Table 2) [36]. In a study of relationships with peers and family, hikikomori were found to have experienced more rejection from peers and parents, had a greater tendency towards shyness, and experienced a higher level of maladjustment to school when compared with university students (Table 2) [37]. The scores of hikikomori compared to university students were 52.83 ± 12.27 versus 46.89 ± 9.76 for shyness, 2.21 ± 0.70 versus 2.09 ± 0.71 for maternal avoidance, 10.29 ± 4.44 versus 7.41 ± 4.02 for parental rejection, 3.85 ± 2.31 versus 2.41 ± 2.15 for peer rejection, and 4.50 ± 1.62 versus 3.20 ± 1.85 for maladjustment to school, respectively (Table 2). Low to moderate correlations at *r* = 0.219–0.400 (*p* < 0.05–*p* < 0.01) with the aspects of shyness, ambivalent maternal attachment, adjustment to middle school, parental rejection, parents threatening a loss of relationship with or ignoring their child, and peer rejection were reported as having been experienced by hikikomori [37]. They further indicate that hikikomori had a low level of connectedness with others due to a lack of support from peers or parents. This, along with their shy temperament, would add to their difficulties in initiating or building relationships.

The types of relationships hikikomori maintained and asocial behaviors they exhibited have also been investigated. In two studies, 19.0- 34.2% of hikikomori were found to have no relationships at all; while 57.4–63.0% still maintained relationships with others, mostly family members, (Table 2) [38, 39]. They exhibited social withdrawal behaviors, with the duration ranging from three months [3] to twenty-five years [40]. In Chauliac et al.’s [38] study, 27.0% of hikikomori did not leave home, while 35.0–38.0% still went on outings, either alone or accompanied. Typical daily activities of hikikomori, in terms of number of hours, were: 7.83 ± 1.99 sleeping, 5.09 ± 4.97 using the computer, 3.11 ± 5.03 using a tablet or mobile phone, and 1.90 ± 1.03 eating, while the remainder of their time was spent watching television, reading comics or animations, reading, idling, or facing the wall (Table 2) [41]. Hikikomori were also reported to have difficulties with interpersonal relationships, social interactions, or fitting into society [42, 43], and experienced peer rejection (Table 2) [37, 42]. In one study, 74.1% of hikikomori had difficulties with interpersonal relationships and social anxieties. For example, 36.2% feared meeting people, 48.3% were anxious about meeting with familiar people, 51.7% were worried about people’s impression of them, and 53.4% could not blend into groups (all *p* < 0.001 when compared with non-hikikomori between the ages of 15–39) (Table 2) [43]. Hikikomori showed disconnectedness with peers and society, had limited relationships and most of those were with family [38, 39], and experienced social anxiety [43], making it difficult for them to establish relationships.

The case studies were of eleven hikikomori, nine males and two females, who had been in social withdrawal for 2–20 years, were aged 13–40, and whose behaviors were reflective of those reported in the above quantitative studies, namely, confining themselves at home, spending the majority of their time in their room, and not engaging in any social relationships or avoiding face-to-face contact with others (Table 2) [44–49] and having social anxieties [50]. One hikikomori felt exhausted from effort in maintaining relationships, was unable to relate well with others, feared entering adult society, and had no confidence in coping with society [51]. In three of the cases, the hikikomori refused to have contact with their family members [46–48] with one hikikomori using furniture to block entry to his room to avoid contact [48]. One of the hikikomori would leave home once a month for appointments at an outpatient clinic [44]. While another reported of mistrusting their parents and inability in approaching the opposite gender when interested [50]. A reversed sleep/wake cycle of being awake in the evening and sleeping during the day was reported [44, 47].

The results from the four qualitative studies were consistent with previous reports of disconnected behavior, which described hikikomori’s losing touch with the outside world, having no peer relationships, or being unable to get along with others [6, 52, 53]. In one case, a mother could not see her child face-to-face for months [6]. These four studies further explored the underlying reasons behind the social withdrawal of hikikomori. Some of the reasons given were a lack of trust in people [52, 53], having experienced a traumatic life event [6, 52, 53] such as bullying, the death of a family member, or the divorce of one’s parents. In a study by Wong and Ying [54], some hikikomori were reported to be conducting intimate relationships online, but had no intention of meeting those people in person. While Chan [55] found, the higher the friendship or intimacy level, the more forms of online communication would be shared between the youth and that peer. Although the dynamics of these online relationships have not been explored, which may indicate a knowledge gap.

### Domain of hope and optimism

Hope and optimism involve a belief in one’s ability to recover, find the motivation to change, have hope-inspiring relationships, think positively and value success, and have dreams and aspirations [24]. In this domain five studies relating to hope and optimism in hikikomori were found, four of which were qualitative studies and one a cross-sectional study.

In the current literature, the belief of individuals in their ability to recover from the hikikomori lifestyle has not been explored. There have been reports on the motivation of hikikomori to change and return to society; however, there were obstacles that they could not overcome, such as feelings of anxiety or their lack of qualifications to list on their resume [2, 56]. Some hikikomori formed positive and inspiring relationships from meeting someone they trusted, received encouragement from someone they knew and liked [2], or met someone on the Internet [53]. In contrast, the hikikomori in Yong and Kaneko’s [57] study were cautious about establishing relationships over the Internet due to fear and an inability to trust people. Hikikomori are seen as people who engage in negative self-appraisals and thinking [56, 57]. They do not talk about success, but rather exude a sense of failure. In two of the five qualitative studies, hikikomori harbored feelings of hopelessness about their future [57]. They thought that they would be unable to secure a job, felt inadequate or incompetent, and complained that society was too demanding and unfair [57]. Hikikomori felt unable to do anything [56, 57] and had a fear of failure [56]. Their dreams and aspirations have not been explored in a qualitative context, however, in a cross-sectional study hikikomori were found to have high scores in the aspect of unclear ambitions about the future. A newly developed scale was used in that study, which measured scores on the unclear ambitions for the future of three participant groups: hikikomori between the ages of 20 and 39, age-matched NEET (people not in education, employment, or training), and working adults; it was found that of the three groups, hikikomori ranked the highest in having unclear ambitions for the future [58].

### Domain of identity

This domain involves the following: the multiple dimensions of identity, rebuilding or redefining a positive sense of identity, and overcoming stigma [27]. The multiple dimensions of identity, as applied towards hikikomori, would be gender, ethnicity, culture, religion, social class, personal identity and attributes, and sexual orientation. In this domain 29 articles were found, including 13 cross-sectional studies; seven case reports, studies or series; one mixed-methods study; one longitudinal study; one pilot case control study; one interventional study; and five qualitative studies.

Of the 13 cross-sectional studies on hikikomori, involving a total number of 1719 hikikomori, the majority were males (1043 (60.7%) to 564 (32.8%) females), although this distribution may be due to the sampling approach used in the studies; one study did not report their gender distributions. By contrast, Wu et al. [42] reported slightly more females (*n* = 90, 53.6%) than males (*n* = 78, 46.4%). More studies are needed to further explore gender distribution ratios and if there are behavioural differences between male and female hikikomori. Hikikomori are not confined to any specific race or nationality, and American, Brazilian, Chinese, French, Italian, Japanese, Korean, Oman, Spanish, Taiwanese, and Ukrainian hikikomori have been featured in cross-sectional studies or case reports (Table 2) [3, 38, 39, 41, 43, 45, 47, 49, 50, 59–63]. No studies were found on the culture or religion of hikikomori; however, hikikomori were reported to have high levels of computer or Internet use, ranging from 5.09 ± 4.97 to 5.20 ± 3.40 h per day [3, 35]. Further investigations may be considered to identify whether long durations of computer use are part of hikikomori culture and to determine what are the cultural norms of hikikomori. Studies of religion and culture may be conducted to uncover more about the phenomenon; however, they would not contribute towards the recovery of hikikomori. Seven studies reported on the dimension of social class. The majority of hikikomori are reported to have a high school level of education or above [36, 40, 42, 63]. However, in two studies it was unclear what educational level they had achieved: in their study, Yong and Nomura [43] reported that the majority of hikikomori had finished school but did not report on their level of education; while Nagata et al. [64] reported the average years of education received being 11.7 ± 1.7, but it is unclear whether the preschool years were included in that figure. Two studies reported that the majority of hikikomori belonged to the middle class [43, 60], while Wu et al. [42] reported that the majority lived in low-income areas. As only a few studies on the social class of hikikomori have been conducted, these might not be representative findings.

With regard to personal attributes and identity, hikikomori were found to have higher levels of passive-aggressiveness, a tendency to adjust their emotions to the environment and people, an inclination to suppress the expression of emotions when feeling shaken in social situations, and to need and be open to emotional relationships [65] when compared to non-hikikomori using the Rorschach Comprehensive System (Table 2). Three qualitative studies reported that hikikomori are lacking of self-esteem or self-confidence [2, 56, 57]. Also reported as features of hikikomori are non-competitiveness, ineffective communication, identity issues because of negative appraisals from people [57], or difficulties explaining themselves to people because they lacked a social identity such as a title to an occupational or student status [56]. A longitudinal study found that hikikomori had low to moderate scores for interpersonal support, belongingness, and self-esteem, with Interpersonal Support Evaluation List scores (ISEL) of 24.60 ± 6.30 out of 48, scores of 6.00 ± 2.45 out of 12 for belongingness, and 5.59 ± 2.08 out of 12 for self-esteem [35], as shown in Table 2. No comparisons were made with age-matched youth without social withdrawal in the study. However, this compared to delinquent age matched Chinese [66] and United States freshman students [67] (26.19 ± 4.38 and 38.30 ± 6.82, respectively) would seem low. This ISEL has not been used normal youth population studies in Asia. No studies were found that explored the sexual orientation of hikikomori.

On rebuilding and redefining a positive self-identity, the previously mentioned studies on personal identity found that hikikomori lack a positive sense of identity [2, 56, 57] and need to rebuild it. In a case study, it was reported that a hikikomori exhibited behaviors of work refusal, felt ashamed of himself and feared of being labelled unemployed [51]. While some hikikomori may use the Internet to find a positive self-identity [53], relationships were also found to be important to their self-esteem [60]. Self-esteem refers to the positive and negative viewpoints individuals have of themselves [68]; and having high self-esteem may result in beliefs of being good, worthy, and positively viewed by others [69], therefore a positive sense of identity. In Chan and Lo’s [60] study, they found that self-esteem was highly correlated with relationships with parents: *r* = 0.73, *p* = 0.0000, siblings: *r* = 0.66, *p* = 0.0000, teachers: *r* = 0.13, *p* < 0.05, and peers: *r* = 0.16, *p* < 0.05, and that the higher the association, the higher was the self-esteem of the hikikomori.

It has been acknowledged that hikikomori are sensitive to people calling them names, such as: “hidden youth” and “withdrawn guys” [70]. However, in the dimension of stigma, only one study was found. The qualitative study, from Japan, found that parents desired to avoid stigma and the label of mental illness, and therefore chose to continue to call their child a hikikomori, even after their child received a psychiatric diagnosis [71]. There have been no other studies on stigma and hikikomori, which suggests that there is a need for more studies to be conducted on this subject.

### Meaning in life

Meaning in life refers to the meaning of mental illness experiences, spirituality, quality of life, meaningful social roles and goals, and the rebuilding of one’s life [24]. As not all hikikomori have a comorbid mental illness, in this study the dimension of the meaning of mental illness experiences will instead refer to the meaning of the hikikomori experience. In this domain a total of 11 articles were found, comprising three cross-sectional studies, five qualitative studies, and three case reports.

In understanding the life experiences of hikikomori, an international cross-sectional study reported that hikikomori experience high levels of loneliness, at 55.4 ± 10.5 out of 80 on the University of California Los Angeles (UCLA) Loneliness Scale (Table 2), in comparison to a score of 40.0 for normal controls found in other studies in these same countries [36]. Five qualitative studies, each with 4–30 subjects selected conveniently from outreach programs, non-profit organizations, or online forums, reported on the lifestyles, feelings, and thoughts of hikikomori. They found a lifestyle centered on confinement at home, with little social contact, and having negative feelings or thoughts [2, 53, 57]. Commonly reported were feelings of low self-esteem, a lack of confidence, hopelessness, and a loss of trust in people [2, 57]. Also reported were of the feeling that no one would care if they died, that they were living like an animal [2], a fear of social interactions [57], boredom, the sense that they could no longer stand staying at home, a loss of interest in computer games [53], and that they did not claim that the hikikomori experience was pleasant [71]. In contrast, some reported feeling freed from restraints and timelines and enjoying their seclusion [53]. Poor hygiene behaviors were reported in a minority of hikikomori [38, 49, 72], with one person defecating and urinating in jars or bottles in his room [49].

No studies were found on spirituality and meaningful social roles or goals in relation to hikikomori. Two cross-sectional studies were found measuring quality of life. One study reported lower quality of life scores, as measured using the Chaban Quality of Life Scale, for hikikomori with or without psychiatric comorbidities, with scores of 11.7 ± 2.70 and 13.7 ± 3.3 respectively, both with a statistical significance of *p* = 0.001, when compared with the control group of non-hikikomori, at 19.3 ± 3.50 (Table 2) [61]. On the contrary, Chan and Lo’s [4] study using the World Health Organization Quality of Life scale, reported an improvement in the quality of life of hidden youth as the time spent being socially withdrawn increased, with an overall correlation of *r* = 0.550; *p* = 0.0000. This may indicate a positive adjustment in the well-being of hidden youth. However, those with a higher degree of social withdrawal were seen to have a lower quality of life, with an overall correlation of *r* = − 0.850; *p* = 0.0000 (Table 2) [4]. This would indicate that social withdrawal is not a positive factor in the quality of life of all hikikomori, and that the severity of the act of social withdrawal could be a factor in whether it is a positive experience. In the dimension of rebuilding life, a qualitative study found that hikikomori had difficulties with stepping out of their room at first and initially felt a sense of insecurity [54].

### Empowerment

The domain of empowerment encompasses personal responsibility, control over life, and a focus on strengths [24]. In this domain five studies were found, two interventional studies, a mixed method study, a qualitative study, and a case study; of which most were focused on interventions for hikikomori. No studies were found that explored the issue of control over life in relation to hikikomori. Studies on how hikikomori experience all dimensions of empowerment are lacking. All interventions exploring empowerment will be discussed in the section on interventions using elements of CHIME.

### Interventions for Hikikomori using elements within the CHIME framework

Intervention, case studies and qualitative studies were found using specific elements within the CHIME Framework, such as rebuilding a positive self-identity, social roles or life, and empowerment through the interventions, namely, Free Space Wood [56], psychotherapy [50, 73], social worker engagement [54, 74], play therapy [75], and C-BED [18].

An ethnographic study was conducted out of a private support group operating out of an alternative school named Free Space Wood in Japan; it reported to offer hikikomori a new social environment to rebuild their social roles and identity [56]. However, the effectiveness of the program was not reported. Of studies exploring the use of psychotherapy to help hikikomori rebuild their lives, showed that a long duration was needed until recovery was achieved, and that it was difficult to convince hikikomori to stay in the therapy program. In the case studies, a duration of two to four years was reported before recovery was achieved [50, 73]. In Lee et al.’s [3] study, 41 hikikomori received a mean number of 2.8 sessions of psychotherapy with home visits. Close to 50% did not show any improvements in their Global Assessment Functioning scores post-intervention, as shown in Table 2. In Hattori’s [73] study, which had a 50% attrition rate, hikikomori were reported to have spent the first six months to one year testing the reliability of the therapist, before rejecting the therapist due to mistrust [73]. This might suggest that the effectiveness of psychotherapy for this group still needs to be evaluated.

The engagement of social workers using empowerment was examined in two studies. In a quasi-experimental intervention study, social workers engaged young people online by identifying their strengths and resources for achieving goals and coping. A portion of the participants were socially withdrawn youth, and the study reported a 4.61% decrease in social withdrawal after the intervention. However, scores from scales measuring such aspects as emotional distress, perceived social support, problem-solving skills, and attitudes towards seeking help were not reported for the socially withdrawn [74]. The exhibited behaviors of high computer usage and histories of social anxieties, may have led health practitioners to attempt to engage hikikomori through the online platform. And a study evaluating forms of counselling to engage hidden youth found using an integrated approach with online and offline counselling showing the highest positive outcomes for total means scores of quality of life (3.74 ± 0.60) when compared to a singular approach of using only one method (online 3.02 ± 0.43; offline 2.52 ± 0.27) (Table 2). Moreover, online counselling was mentioned to offer a platform for communication, while offline counselling offered opportunity for mediation during conflicts between youth and their family [76] although the study did not investigate or report on recovery [76]. A qualitative study reported that social workers enabled socially withdrawn youth to reengage with the outside world by accompanying them to outings to provide a sense of security [54]. And behaviors of feeling insecure when reentering society with sudden setbacks or refusal of social worker re-engagement; therefore, recovery would need to be at the pace of the user and would be a non-linear process [54]. Although there were no reports of recovery, this approach might be beneficial for such youth.

Two other intervention studies and a case study were also found focusing on the use of empowerment and a strength-based approach. An interventional study by Chan [75], which investigated whether play therapy with online games would empower and improve the psychological well-being of hidden youth, reported significant correlations between play therapy and empowerment, with *r* = 0.59; *p* < 0.05, and the individual’s positive psychological state of development, at *r* = 0.600; *p* < 0.05. A hierarchical regression analysis has further suggested that empowerment is a strong predictor of the positive outcomes of play therapy. In a pilot intervention study called C-BED, an online platform was used to empower peer-to-peer shared learning and dialogue with online modules. The five hikikomori who participated exhibited a decrease in anxiety and an increased willingness to participate socially; however, no measurement scales were used in the study [18]. A case study of one hikikomori using strength-based coaching reported that the subject had returned to school and showed an improvement in scores in the Rosenberg Self-Esteem Scale and Subjective Vitality Scale, and a decrease in score in the Kessler Psychological Distress Scale following the intervention. The results of the individuals from pre-intervention to post-intervention were 16 to 25 (scoring 0–30), 1.8 to 3.4 (scoring 0–4), and 17 to 6 (scoring 10–50), respectively [77]. Although improved psychological well-being was observed from a strength-based focus, testing still needs to be conducted using a much larger sample.

All of the interventions mentioned may be possible options for recovery, but further evaluation is needed since many of the studies had small sample sizes or did not have a comparable comparison group, and some did not report on the effectiveness of the intervention. Researchers have identified factors such as the internet [53] and having relationships [60], giving hikikomori a positive sense of identity or better self-esteem, these may be areas that interventions could be designed to target. However, more studies on rebuilding the positive self-identity of hikikomori are needed to better understand the phenomenon and to determine whether more factors are involved. Further explorations into interventions are needed as the mechanism to recovery is still unknown. Examples would be from a one-year longitudinal study that was not an interventional study with a resulting increase in social connectedness over time (SNI from 2.79 ± 1.80, 2.93 ± 2.06, to 3.09 ± 1.87) (Table 2), and to a level of recovery that was sufficient to allow a return to the workforce of almost 50% of the hikikomori in the study [35]. These results arose from an unintentional intervention administered by social workers during home visits to hikikomori during the period of the study, which involved the use of archived patient records [35]. However, in an interventional study of a home visitation therapy program offered by psychiatrists to hikikomori over a period of one year, no statistically significant difference was seen in any of the outcome variables of their study, which included social networking [39]. This suggests that further exploration is needed to understand what mechanisms help to improve the ability of hikikomori to connect with other people.

## Discussion

**Fig. 2:**
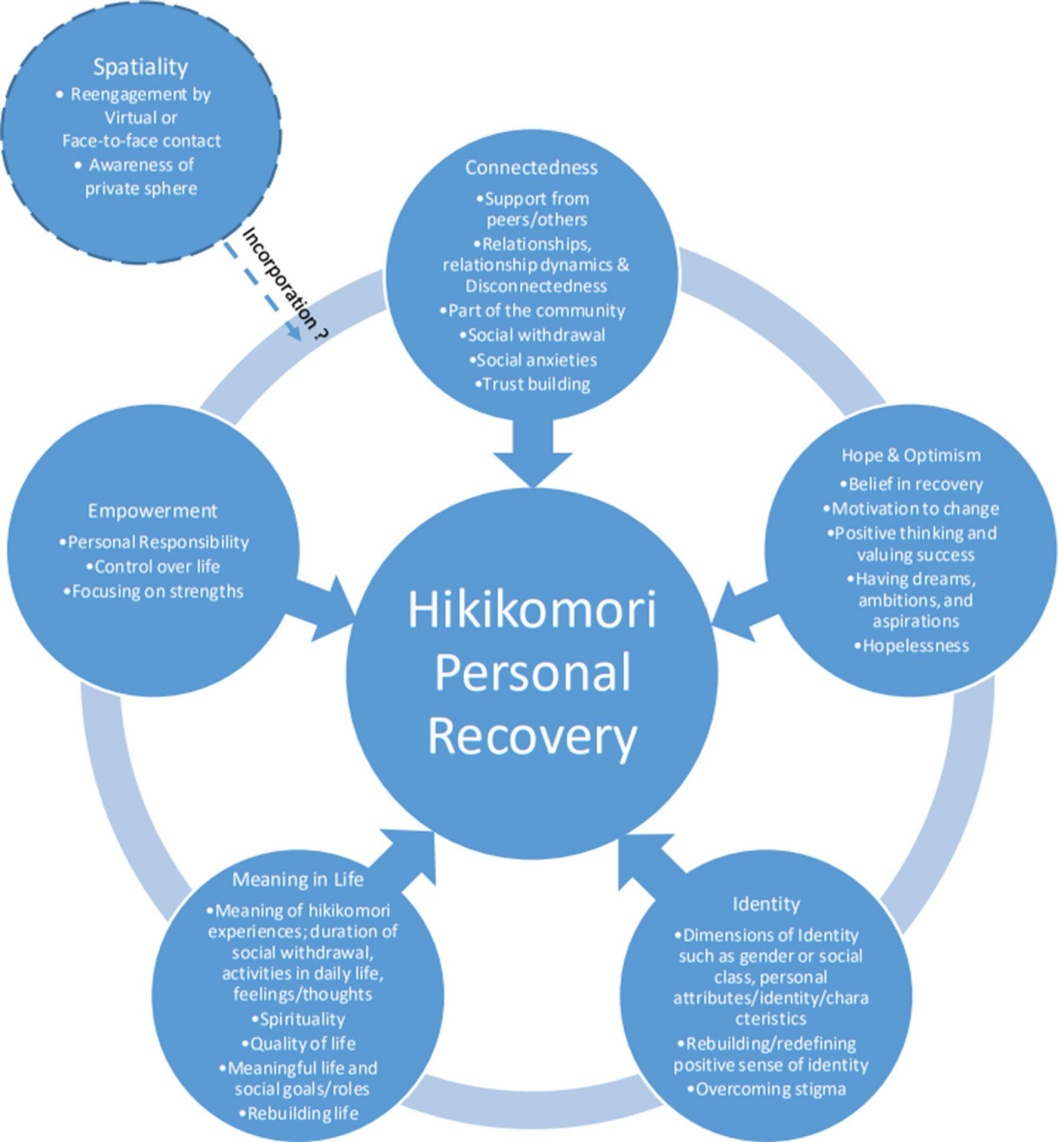
Modified CHIME framework for personal recovery in Hikikomori Care

The CHIME framework for personal recovery was applicable to understanding the life experiences of the hikikomori population. Forty-four studies were reviewed, most of which were quantitative in nature (75%) with close to one third being cross-sectional studies (29.5%). To apply this framework to hikikomori, slight modifications were made to the framework, including the domain of identity. The multiple dimensions of identity were expanded to encompass gender, religion, social class, personal identity, and attributes. In the domain of meaning, the meaning of the illness experience was replaced by the meaning of the hikikomori experience. Modifications to the framework are displayed in Fig. 2.

Thematic overlap occurred between some domains, such as connectedness, identity, and meaning. The identity of being a hikikomori was defined in relation to the hikikomori’s lack of connectedness with society and lack of meaningful social roles; however, this did not affect the application of the framework. By using the framework to assess the literature, hikikomori were shown to be disconnected from peers and society, to have relationships limited mostly to family, to experience social anxiety, engage in negative self-appraisal, feel a lack of identity, exhibit a mistrust of people, and have high levels of passive aggressiveness and a shy temperament. Those leading a hikikomori lifestyle were not limited to a specific race. More studies with a larger sample size need to be conducted to determine differences in gender distribution and social class. Although quality of life seemed to increase with time spent in withdrawal, higher levels of social withdrawal led to a lower quality of life.

The CHIME framework was able to encompass most of the aspects relating to hikikomori. However, the limitations of the framework were its inability to address the building of trust and the characteristic of non-linearity in the recovery process. For hikikomori, building trust is an important stage in their recovery process [54, 73]. Long durations of six months to a year spent in building rapport have been reported [73]. Without the establishment of rapport, the recovery process will not begin. The building of trust could be incorporated as a dimension in the CHIME framework for hikikomori. The term “yo-yo process” has been used to describe the process of recovery for hikikomori and the setbacks that they experience, such as a reversal in their progress [54] or their sudden refusal to take part in social activities or have face-to-face contact [6], which has been reported by social workers during reengagement work. This indicates that the recovery process is not linear or a matter of taking one step after another, but rather is a non-linear process. For hikikomori, non-linearity can occur at different levels and phases of recovery; however, this aspect is not apparent when using the CHIME framework and needs to be incorporated. An additional limitation of the framework is the inability to address aspects of spatiality in hikikomori care. Due to the self-secluding nature of hikikomori, the majority of care is currently provided in the home setting and starts where the client is [54, 70]. Home visits require sensitivity and awareness [6] from the healthcare team, since they are entering into each of the clients’ private sphere. Sensitivity to each client’s surroundings can provide clues about his or her hobbies or interests, which can be used as the base for initiating interaction and discussion for clientele reengagement [6]. The reengagement process can also help show recognition to the clients in view of their need for privacy [6]. Spatiality is a component specific to hikikomori reengagement which without the clients can hardly be outreached at their homebound comfort zone in the first place; however, it is not a dimension or domain in the CHIME framework. Consideration has to be given for accommodating the special needs of hikikomori. In addition, in the CHIME framework there is no differentiation of the level of importance of each dimension. With regard to hikikomori care, two components are of great importance: relationship dynamics and activity (type or level). These two components can be used to measure the level of connectedness and distinguish the progress in the recovery achieved by individuals. Both categories lead to the ultimate goal, which is to reconnect with society. Consideration has to be given for incorporating the domains of relational dynamics and activity into the CHIME framework, or for adopting them as major sub-domains under the domain of connectedness.

The CHIME framework is applicable to focus on studying the psychosocial aspects of the hikikomori lifestyle and to identify areas in hikikomori research studies that have not been explored. A summary of the applicability of hikikomori research and of the areas that are yet to be studied is provided in Table 4. Studies on hikikomori in relation to the CHIME framework were found predominantly in the domains of Connectedness (the majority, totaling 22 out of 44 studies), Identity, and Meaning in Life; however, literature is lacking on the domains of Hope and Optimism and Empowerment. One possible direction of research in the future is to focus on exploring aspects of these domains to understand what could be of benefit to hikikomori. This may lead to recommendations or interventions helpful for hikikomori care. Further work is needed to clarify details on the sociodemographic and ethnographic characteristics of hikikomori such as their gender distribution, gender differences, sexual orientation, culture, and religion; to understand the dynamics of the intimate relationships of hikikomori through qualitative research; to understand the meaning of recovery for a hikikomori and what enacted and felt stigmas would be present through taking a qualitative approach; to understand what motivates hikikomori to work towards recovery or what they value through taking a qualitative or mixed-methods approach; to understand what the meaningful social roles or goals are for hikikomori and how they establish them through a qualitative enquiry that may lead to interventional designs; to understand the meaning of control over life and personal responsibilities for hikikomori through qualitative research; to explore more aspects of empowerment to aid their recovery; to understand what measures can to be taken to help hikikomori overcome their sense of failure or fear of failure rather than valuing success defined in terms of climbing up the social ladder, to help them towards recovery, to determine what measures need to be taken to improve this situation and whether they would help towards recovery; and to figure out what components of future interventions may be useful for recovery and welcomed by hikikomori. A further exploration of these areas could lead to greater understanding and improve hikikomori care.

## Limitations

A known limitation to this study was by using the search formula as per Li and Wong’s [9] systematic review, there may be the risk of missing articles; which was mentioned in their study due to the formula omitting the search terms of “social isolation” and “non-engaged”. However, the search terms used according to the domains of CHIME were able to locate a substantial amount of publications exploring the hikikomori phenomenon prior to exclusion process. A second limitation to this study was from confining the search of relevant publications to the English language but excluding those in Japanese in particular, where the phenomenon of hikikomori started to take place; thus risking the chance for missing hikikomori research of publications in languages other than English. A third limitation was due to the busy schedules and time conflicts between team members, and the logistics when screening of articles, it did not allow for a useful Kappa coefficient to be produced. Lastly, trial registration of this review was not completed; as the review was in the stage of dissemination of findings when understanding that registration into the registry required completion before any synthesis of data.

## Conclusion

The CHIME framework is applicable to the hikikomori population and can encompass most aspects of their life experiences; however, future modifications may be needed to include the three major domains of spatiality, relational dynamics, and activity; as well as the dimensions or sub-domains of trust building and non-linearity. Through the use of the framework, the hikikomori lifestyle is shown to be characterized by: disconnection from peers and society, limited relationships largely confined within the family, social anxiety, negative self-appraisal, a lack of identity, a mistrust of people, a high level of passive aggressiveness, and shyness. Hikikomori are not confined to a specific race, and higher levels of social withdrawal would tend to lead to a lower quality of life. Using the CHIME framework, many gaps in knowledge about hikikomori could be identified in the literature, such as those about gender distribution, behavioral differences between males and females, sexual orientation, culture, religion, and the dynamics of intimate relationships; the meaning of recovery for hikikomori, the impact of stigma on them, and how they can be motivated towards recovery or into taking up valued, meaningful social roles or goals; the meaning for hikikomori to have empowerment and control over their life and personal responsibilities; and any other components of future interventions considered useful and welcomed by hikikomori. More understanding of these issues is needed to improve hikikomori care. Considerations could be made for future use of CHIME in hikikomori care, as individuals may be entrapped in the lifestyle for long periods of time [44] and recovery has been reported to take a minimum of two years [46, 73]; the framework could possibility target areas that would need attention or improvement during the time of entrapment in the lifestyle. However, the full applicability of the CHIME framework towards recovery from the hikikomori lifestyle would need further verification; further studies with recovered hikikomori could verify if the domains of CHIME were involved towards their recovery.

## Data Availability

All data generated or analysed during this study are included in this published article.

## Abbreviations

CHIME: Connectedness, Hope and Optimism, Identity, Meaning in Life, Empowerment
PRISMA: Preferred Reporting Items for Systematic Reviews and Meta-Analysis
JBI: Joanna Briggs Institute
MMAT: Mixed Method Appraisal Tool NEET: not being in education, training, or employment
SNI: Modified Berkman–Syme Social Network Index
ISEL: Interpersonal Support Evaluation List scores
UCLA: University of California Los Angeles
vs: Versus
WHOQOL-BREF: World Health Organization Quality of Life scale-brief
QOL: Quality of life
C-BED: Community Based Enterprise Component
LSNC: Lubben Social Network Scale
GAF: Global Assessment Functioning Scores
N/A: Not applicable
